# On Rheumatic Pericarditis

**Published:** 1855-09

**Authors:** 

**Affiliations:** Coblentz


On Rheumatic Pericarditis. By Dr. Eutenberg, of Coblentz. —From experience collected by the author, the following conclusions are deduced. *First*, as to the symptomatology; most authors affirm that there is pain either confined to the region of the heart, or extending from the left side to the shoulder and the mesogastrium. Dr. Eutenberg found it in none of his cases; and he adds that Lænnec, Hope, and Bouillaud, have all remarked upon its occasional absence; hence he infers that it is a sign of complication with pleurisy or pneumonia. At a later period, when abundant exudation is poured forth, a heavy pain is experienced.

Severe feverish symptoms occur only in the very acute cases. Then the thirst is oppressive; but the power of drinking limited on account of the severe dyspnœa. The urine is mostly very red, with a sediment of phosphate of ammonia and uric acid. There are severe night sweats, especially about the head.

At the commencement there is a marked sense of oppression, which becomes worse with the increase of the exudation.

The position of the patient is characteristic, whether the pericarditis be acute or chronic. He avoids lying on the left side. The recumbent posture on the back, or, if there be pleurisy, the sitting posture are most convenient. There is generally cough, at first of catarrhal character. Sickness, as mentioned by Knox, Creysis, and Bouillaud, Dr. Eutenberg found only once in eight cases. The pulse is very quick, 140—200. The sounds of the heart are mostly clear and defined; but a peculiar fluttering movement accompanies the heart's beat, when the exudation is excessive, and interferes with its action. Hope remarked this in connection with carditis. The pulse is always small, but not intermitting. Respiration is hurried, but without remission. There is no sign of adhesion of the pericardium to the heart. The author found it twice in bodies where he could not diagnose it during life.

The author did not find Rokitansky's assertion confirmed, that in the neighborhood of an abscess of the heart, the muscular substance is infiltrated with pus, easily lacerated and discolored. The want of redness in the pericardium he found remarkable.

The treatment must depend upon the form of disease. In acute cases, attacking muscular subjects, a moderate venesection is recommended; but not its repetition, as Bouillaud has advised. In carditis no bleeding, and in endo-carditis very moderate abstraction of blood is recommended. The author makes chief mention among internal medicines of tartarized antimony, combined with sulphate of magnesia, or calomel in severe cases. Should the pulsations of the heart and shortness of breath be considerable, and combined with hurried pulse, Dr. Eutenberg gives Corros. Sub., gr. j, dissolved in Sp. Vin. Rectif., ℥iij. Three to five drops to be taken twice daily. Next to corrosive sublimate stand the preparations of gold, but they are less fit for acute cases. In chronic relapsing pericarditis the iodide of iron is recommended.—[*Pr. Ver. Ztz.*] *Half-Yearly Ab. Med. Sciences.*

